# Early trifurcation of the facial artery including a dedicated buccinator branch: anatomical and surgical implications

**DOI:** 10.1080/23320885.2026.2664264

**Published:** 2026-05-11

**Authors:** Niccolò Fagni, Ferdinando Paternostro, Guglielmo Maria Fiori, Ludovica Livi, Giorgio Zinno, Jacopo Junio Valerio Branca, Eugenio Bertelli, Immacolata Belviso

**Affiliations:** ^a^ Department of Medicine, Surgery and Neuroscience, University of Siena, Siena, Italy; ^b^ Department of Experimental and Clinical Medicine, University of Firenze, Florence, Italy; ^c^ Department of General Surgery, Federico II University Hospital, General Surgery Resident, Naples, Italy; ^d^ Department of Molecular and Developmental Medicine, University of Siena, Siena, Italy; ^e^ Department of Psychology and Health Sciences, Pegaso University, Naples, Italy

**Keywords:** Facial artery, anatomical variation, early trifurcation, premasseteric branch, buccinator branch, facial artery musculomucosal (FAMM) flap, cadaveric dissection, angiographic imaging, reconstructive maxillofacial surgery, iatrogenic injury prevention

## Abstract

Variations in the branching pattern of the facial artery may affect the planning and outcome of reconstructive and aesthetic procedures in the midface. Here, we report a rare anatomical variant in which the facial artery trifurcates early into three distinct branches, including a novel buccinator branch. In January 2025, the right-sided facial artery of a 67-year-old male Caucasian cadaver, injected with colored acrylic resins, was dissected at the ICLO Teaching and Research Center (Verona, Italy). Following identification of the external carotid artery, the facial artery was traced via an oblique submandibular approach. Branching patterns were documented through digital photography and schematic illustration. Instead of the classical singular course, the facial artery formed a common trunk that, 2 cm distal to its origin, trifurcated within a 1 cm span along the inferior mandibular margin. The anteromedial branch followed the standard facial artery trajectory; the posterolateral (premasseteric) branch supplied the masseteric region; and the central (buccinator) branch penetrated the buccinator fascia, arborizing extensively within the muscle belly. This buccinator branch exhibited a markedly dense microvascular network not previously described. This variant expands the known spectrum of facial arterial anatomy. While the direct clinical impact of a single observation remains limited, awareness of such configurations may be relevant in selected surgical contexts, particularly in flap planning and in procedures involving the buccal region. Unlike the classical buccal artery arising from the internal maxillary system, this branch originated directly from the facial artery and appeared to provide predominant intramuscular vascularization of the buccinator. Further bilateral cadaveric and imaging studies are necessary to determine the prevalence and clinical relevance of this and related anatomical configurations.

## 1. Introduction

The study of the anatomy of the facial artery is paramount in understanding its standard course and the possible anatomical variants. As a critical vessel supplying the face with oxygenated blood, the knowledge of the structure of the facial artery is essential for various medical disciplines, particularly in some surgical procedures. The facial artery perforator flap is a safe and cosmetically advantageous option not only for the immediate repair of oncologic defects, but also for the reconstruction of defects related to head and neck cancers, trauma and congenital conditions. Variations in the facial artery can significantly impact surgical outcomes and patient safety, and aesthetic results, necessitating a thorough understanding of these potential anomalies. Consequently, this introductory exploration serves as a foundation for examining the complexities of the facial artery and its clinical implications [1]. The facial artery arises as a collateral branch of the external carotid artery (ECA), distal to the origin of the superior thyroid, ascending pharyngeal and lingual arteries, and anterior to the occipital artery. In this case, the facial artery arises independently from the ECA. However, due to the dissection technique employed, it is not possible to determine whether this also applies to the superior thyroid artery and the lingual artery. Consequently, it can at least be excluded that the described variant falls within a type III ECA morphology with respect to the origin of the facial artery [2]. It appears immediately behind the lower border of the posterior belly of the digastric and stylohyoid muscles; from there, it takes an initially oblique course upward and forward, deep to the platysma within the superficial cervical fascia. After giving off its collateral, the facial artery passes medially to the lower border of the mandible and crosses the antegonial notch, where its tortuous, looping path allows it to lengthen during chewing movements. Having crossed the mandibular margin and arched over the masseter muscle at its insertion, the artery enters the lower facial region, progressively moving toward the skin’s surface. At first, it ascends toward and posterior to the modiolus (located just near the mouth’s angle and serving as the insertion point for the elevator and depressor *anguli oris*, the zygomaticus major and minor, risorius, and mentalis muscles), then becomes more superficial as it courses toward the nasolabial angle. From there, it continues subdermal at an oblique upward angle along the dorsum of the nose by dividing into its angular and lateral nasal terminal branches [3].

Along its course, the facial artery lies in intimate relation to numerous structures: in the cervical segment, it sits medial to the sternocleidomastoid muscle behind the posterior belly of the digastric muscle, wrapping around the posterior margin of the submandibular gland before emerging superficially, giving glandular branches to the submandibular gland and the submental artery, one of its most consistent branches; higher up, at the inferior border of the mandibular body (antegonial notch), it consistently crosses and lies deep to the terminal branches of the facial nerve; in the labial region, it runs alongside the superior and inferior labial nerves. Parallel to it runs the facial vein, which follows a more lateral and concave course, keeping distance from the artery in the lower face and becoming nearest in the mid-face.

On the face, the facial artery is typically located anterior and medial to the facial vein, which follows a straighter and more lateral course. The distance between these vessels varies along the facial segments, being greater in the lower face and progressively decreasing toward the midface.

The vascular territory of the facial artery includes the mimetic musculature, the submandibular gland, the mucosal and cutaneous structures of both lips, and the medial portion of the cheek and nose. From its principal branches (the labial, tonsillar, ascending palpebral and nasal branches), an extensive network of anastomoses arises with branches of the internal carotid artery (especially the ophthalmic), ensuring critical collateral circulation for the perfusion of the orbit and face [4]. This dual functional and surgical significance makes the facial artery an indispensable landmark in reconstructive microvascular surgery and in the design of pedicled fascio-cutaneous flaps, yielding optimal aesthetic and functional outcomes even in the repair of defects of the oral cavity and oropharynx [5].

The facial artery represents the main vascular pedicle for several reconstructive flaps widely used in head and neck surgery, including the facial artery musculomucosal (FAMM) flap, dpFAMM flap, island FAMM flap, nasolabial flap and submental flap [6,7].

Furthermore, vascularization from the facial artery extends to the myloglossus muscle, highlighting its role not just in facial perfusion but also in contributing to the vascular supply of critical lingual structures [8]. Conventional angiography has proven to be an innovative technique for the study of facial artery variants [9].

This study documents and reports an unusual anatomical variation in the facial artery characterized by a new branch never described before with a peculiar different vascularity, observed during an anatomical dissection of an injected human cadaver specimen.

## 2. Materials and methods

The observations in this case report were performed at the ICLO Teaching and Research Center (Verona, Italy), an authorized institution for cadaveric studies, in January 2025. Written informed consent was obtained from the body donor prior to death for the use of donated tissues for educational and research purposes.

The dissection of a human Caucasian male cadaver aged 67 years, injected with synthetic resins (acrylic polymers) coloured red for arteries and blue for veins, was performed for teaching purposes. The dissection required careful identification of anatomical landmarks and meticulous handling of the artery and adjacent structures. The artery was located using key anatomical landmarks.

The dissection followed a systematic approach: identification of the ECA, tracking of the facial artery from its origin, and detailed documentation of its course and branching patterns. Observations were recorded via digital photography and schematic illustration, with particular attention to variant anatomical presentations.

The specimen was positioned in a lateral decubitus posture, with the dissected side facing upward and the neck slightly extended. An oblique skin incision was made immediately inferior to the mandibular angle, extending along the nasolabial fold. The chosen oblique submandibular approach allowed optimal exposure of the cervical and early facial segments of the artery. The skin and subcutaneous fat were elevated en bloc to expose the superficial musculo-aponeurotic system (SMAS) and platysma. A transverse incision through the investing layer of deep cervical fascia (located between the posterior belly of the digastric and the stylohyoid muscles) revealed an early trifurcation of the facial artery into three distinct branches, each separated by approximately 1 cm at the inferior mandibular border.

The most posterior branch was traced beneath the masseteric fascia into the deep surface of the masseter, where fine muscular twigs were visualized by the red acrylic contrast. The intermediate branch was followed obliquely forward, passing deep to the buccinator and under the buccal fat pad; careful blunt dissection of surrounding connective tissue confirmed its intimate relationship with the buccinator muscle.

The anterior branch preserved the classical course of the facial artery: it looped over the mandibular margin with slight tortuosity, coursed superficially across the masseter, and emerged beneath the SMAS along the nasolabial crease to give off the superior labial, angular and lateral nasal branches.

The contralateral side was not dissected; therefore, bilateral symmetry of this variant could not be assessed.

## 3. Results

During the dissection classes, professors and technicians identified a notable variant of the facial artery and recognized the importance of highlighting this finding through anatomical dissection.

Under normal conditions, the facial artery typically emerges from the ECA near the angle of the mandible, passing deep to the submandibular gland and curving anteriorly and superiorly across the mandible without any added branches. It commonly gives off inferior and superior labial arteries, a lateral nasal artery, and terminates as the angular artery near the medial canthus. In the presented case, however, three distinct branches were identified with different courses and peculiar vascularity.

**Figure 1.**
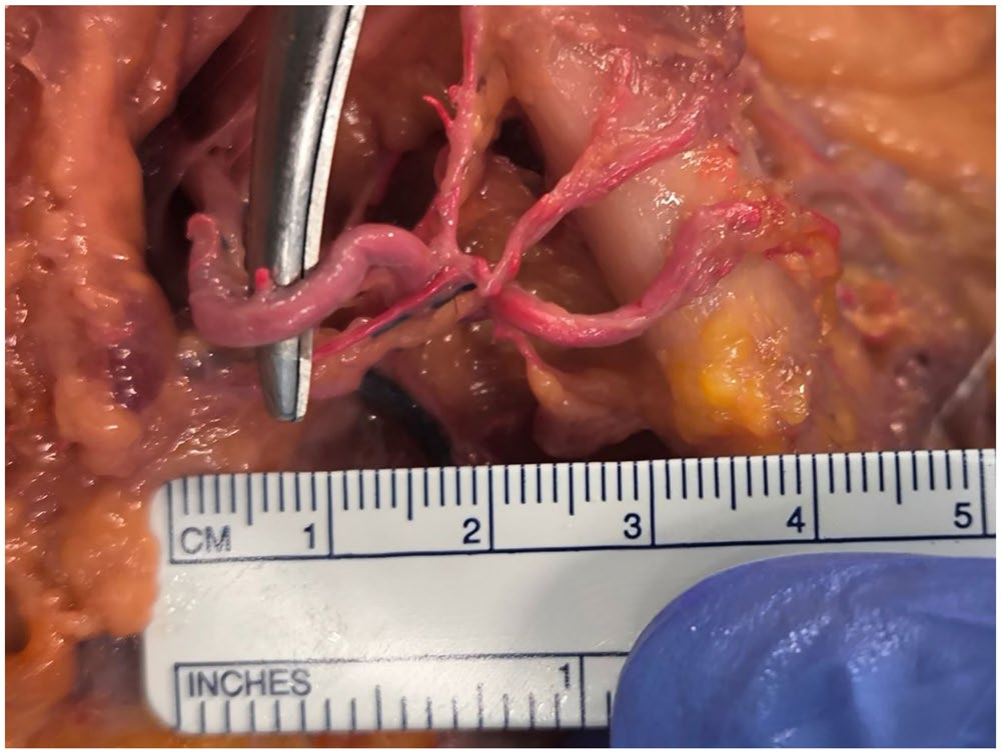
The origin of the facial artery branches. The main facial artery branches are given by the common facial artery after 2 cm from its origin, as clearly reported by the ruler.

In this distinctive anatomical variant, the facial artery first gives rise to the submandibular branch before diverging markedly from its classic textbook course. Rather than continuing as a single vessel, it forms a unified common trunk that, at approximately 2 cm distal to the origin, trifurcates into three well-defined branches (Figure 1).

**Figure 2.**
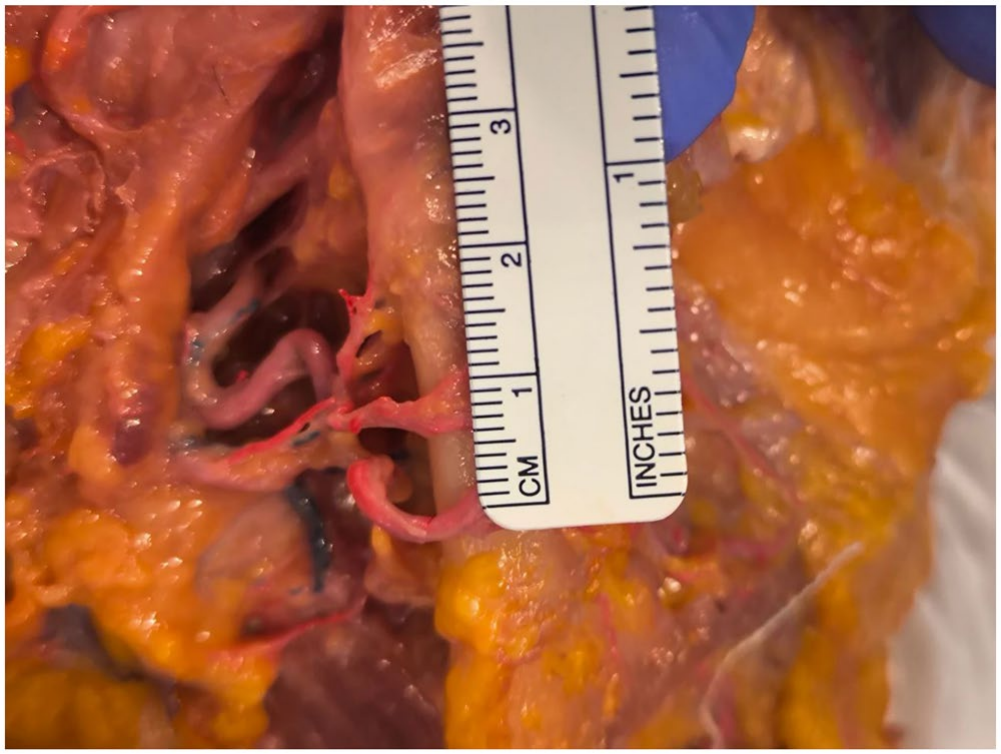
The distance between two facial artery branches. The image shows that the main facial artery branches given by the common facial artery are distanced at the lower border of the jaw line 1 cm from each other.

These branches emerge within a span of roughly 1 cm along the inferior margin of the mandible, creating a compact yet demarcated distribution pattern (Figure 2).

**Figure 3.**
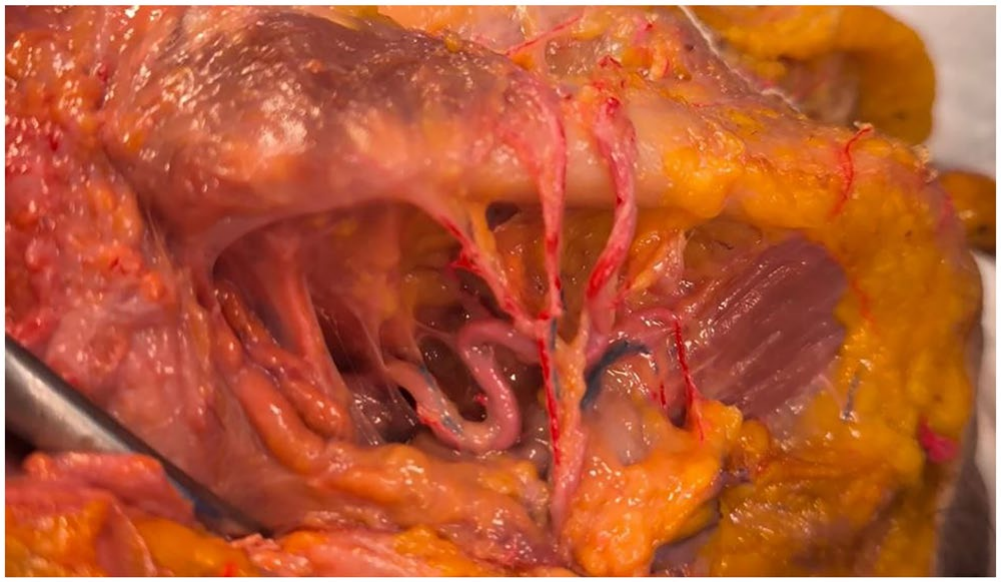
The common facial artery. The illustrated photograph clearly shows the submandibular artery and the common facial artery, which gives off three distinct branches: the most lateral branch, known as the premasseteric or posterior facial artery when present; the central branch, an entirely novel variant that terminates at the buccinator muscle, supplying it; and the anterior branch, identified as the proper facial artery. The anterior branch is individualized as the proper facial artery; notice that it follows the typical tortuosity of the facial artery itself as a continuation of the common facial artery represented in the picture. It is, as usual, also upon the antegonial notch.

The most anterior of these branches retains the identity of the facial artery proper: it ascends along its customary pathway, displaying the conventional caliber, wall structure and branching behavior described in standard anatomical texts. Posteriorly, the second branch (termed the premasseteric or posterior facial artery) courses toward the masseteric region, running superficially to the muscle fibers and supplying vascular inflow to the adjacent soft tissues as noted in earlier cadaveric studies (Figure 3).

**Figure 4.**
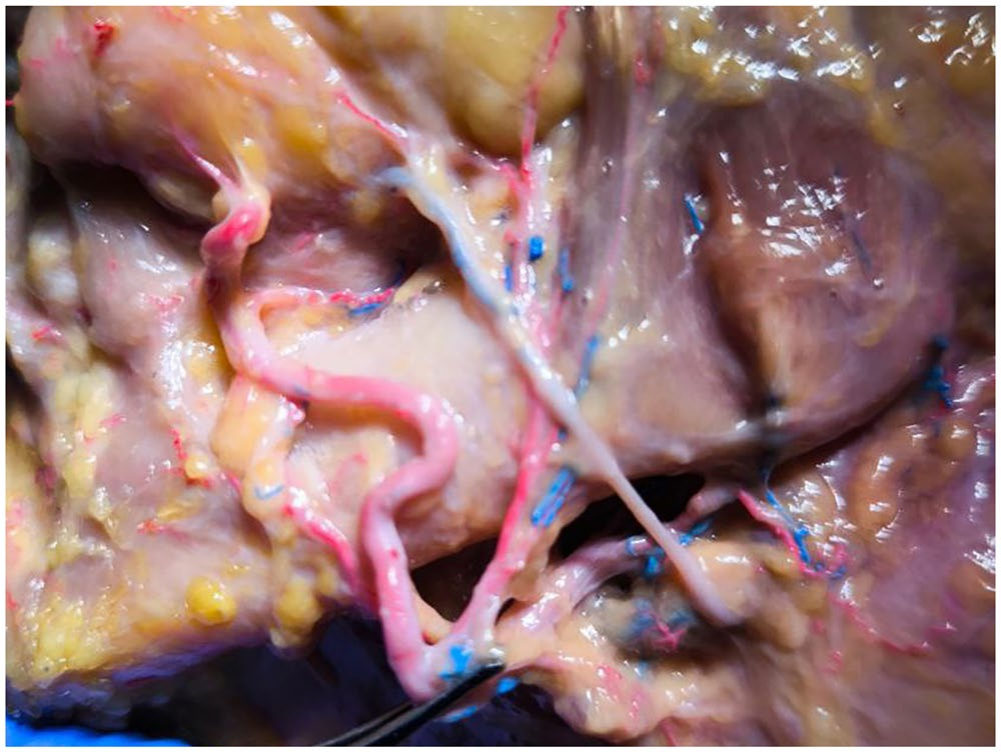
The facial vein pathway. The image highlights that the facial vein is crossing the central branch of the common facial artery arising buccinator vascular territory under the buccal fat pad.

What sets this variant apart is the central branch, here designated the buccinator branch. This vessel departs from the parent trunk with an almost perpendicular trajectory, penetrating the buccinator fascia to enter the muscle belly. It then arborizes extensively within the buccinator, furnishing it with a rich microvascular network (characterized by a dense intramuscular plexus with multiple fine-caliber branches) that appears both morphologically and quantitatively more pronounced than the typical subsidiary branches seen in the classic arterial pattern arising from the internal maxillary artery. The exclusive supply of this muscle by a dedicated branch of the facial artery has not, to our knowledge, been previously documented (Figure 4).

Taken together, these findings expand the known variability of facial artery branching and may have potential relevance in surgical planning of the cheek region, particularly in flap design and in minimizing iatrogenic injury during aesthetic and reconstructive procedures.

## 4. Discussion

It is important to acknowledge that the present observation is based on a single cadaveric specimen. Therefore, its immediate clinical relevance should be interpreted with caution. The majority of reconstructive and oncologic procedures are routinely performed without encountering such a configuration. However, isolated anatomical variations may still be of interest, particularly when they involve atypical vascular supply to functionally relevant structures such as the buccinator muscle.

Anatomical variations of the facial artery have both anatomical and potential clinical relevance. Research has demonstrated that common variations in the branching of facial artery branches can impact anatomical presentation, emphasizing a need for a thorough understanding among clinicians [10]. The ECA, from which the principal arterial branches supplying the cranial splanchnocranium and the surrounding soft tissues arise, is an arterial vessel characterized by distinct morphological features and by an intrinsic variability greater than would be expected in an arterial district. Its complexity is further highlighted by the numerous possible patterns of presentation. Variability involves not only its collateral branching but also its terminal configuration [8]. Broader morphological studies should, in the future, aim to identify functional correlations among the individual variants observed, in accordance with the concept of anatomical fractality [11].

For instance, a study from Mohsen and coworkers [12], which documented different configurations and classifications of the anterior branches of ECA, found that variations in the origins of the anterior branches of ECA occur with notable frequency, with type I variations (superior thyroid artery, lingual artery and facial artery originate from separate branches) encompassing 82% of cases, while less prevalent types account for a small fraction of observed configurations.

In our study, the facial artery has an origin compatible with type I in relationship with the ECA; however, the present study may have the limitation that only one side has been observed.

The striking feature of this variant, however, concerns the presence of three distinct branches after a common facial arterial tract: the premasseteric artery, the facial artery proper and a buccal branch for the buccinator muscle.

Anatomical variants of the facial artery are sparsely documented in the literature. Recent case-based clinical anatomical reports have emphasized the considerable variability of facial artery branching patterns and their potential impact on reconstructive and aesthetic surgical procedures, highlighting the importance of preoperative awareness of such variants to reduce ischemic and iatrogenic complications [13].

Although the premasseteric branch (also known as the posterior branch of the facial artery) has been reported [14].

Three distinct branches at an early trifurcation, including one specifically supplying the buccinator muscle, have not been clearly described in the available literature.

The facial artery is prone to numerous variations, which makes the identification of the variations vital to clinical practice, particularly for orofacial and rhinoplasty surgery. Conventional angiography was used, as it is a vital assessment tool that helps in the assessment of variations in the facial arteries and is suitable for evaluating smaller vascular anatomy, due to the perfect spatial resolution and portrayal of vascular anatomy. Thus, rather than the normal ending of the facial artery as an angular artery, Alharbi disclosed that in certain instances, the artery termination took the form of a superior labial artery with a small lateral nasal artery branch located closer to the midline compared to the normal cases. Regardless of the infrequency of such variations, they may be considered in selected cases, particularly when preoperative imaging is available or when operating in the buccal region [15].

No variation for the terminal branches of the facial artery in this case report was observed, but a particular branch to the buccinator muscle shortly after the origin of the facial artery itself was documented.

Among the collateral branches of the internal maxillary artery is the buccal branch, which arises posterior to the lateral pterygoid muscle and travels between the buccinator and masseter, supplying the buccinator muscle and adjacent mucosa. In the present case, it was not possible to clearly determine the presence of the classical buccal artery arising from the maxillary artery. Therefore, the buccinator branch originating from the facial artery may represent either an additional source of vascularization or a variation in which the typical maxillary contribution is reduced or modified [16].

In our case report, the buccal branch for the buccinator muscle had been identified by observing its distribution territory as a collateral branch of the facial artery, with potential relevance for surgical planning and flap safety.

Moreover, the connections between the facial artery and structures such as the myloglossus muscle further emphasize its importance. As noted in a study conducted by Belotti and coworkers, vascularization of accessory muscles can affect surgical outcomes and the management of conditions affecting the oral cavity [8]. Such findings underscore the importance of considering these variations during surgical planning and interventions to mitigate the risk of complications. Understanding these variations is integral to both anatomical education and clinical practice.

A relevant flap in reconstructive surgery is the FAMM flap. FAMM is a versatile axial flap based on the facial artery, harvested from the inner cheek. It includes buccal mucosa, submucosa, and part of the buccinator muscle, and can be oriented superiorly or inferiorly depending on the reconstructive need. It is widely used for intraoral defects. A recent review highlighted its success across various head and neck reconstructions, confirming its role as a preferred flap in oral cavity surgery [17].

From an embryological perspective, this variant may reflect persistence or hypertrophy of an anastomotic channel between the facial and maxillary arterial systems.

Unlike the classical buccal artery of maxillary origin, the present branch showed a direct intramuscular course and exclusive supply, without evidence of a dominant maxillary contribution.

The facial artery may be considered a highly variable vascular system rather than a strictly predictable vessel. This anatomical variant in the present case report could have significant implications for the efficacy of this procedure.

## 5. Conclusions

This study describes a rare branching pattern of the facial artery characterized by an early trifurcation into three distinct branches: the premasseteric branch, the buccinator branch, and the main facial branch. The discovery of the buccinator branch (which penetrates directly into the fascia and belly of the buccinator muscle, providing an unusually rich microvascular supply) expands the spectrum of known variants and underscores the importance of careful preoperative assessment with angiographic or ultrasound imaging in reconstructive and aesthetic procedures of the facial region.

This variant may be relevant in selected surgical contexts, particularly in the planning of arterial-based flaps, such as the FAMM flap, as well as surgical approaches to mandibular and buccal dissections, where it may contribute to reducing the risk of ischemia or iatrogenic injury.

Finally, a larger-scale collection of data (integrating advanced imaging techniques and bilateral cadaveric studies) is warranted to determine the prevalence of this and other possible configurations.

This finding reinforces the concept that the facial artery should be regarded not as a single vessel, but as a modular and adaptive vascular system of the face. Moreover, our finding emphasizes the need for comprehensive anatomical knowledge of the facial artery and its variants. Cadaveric studies remain indispensable for education and surgical planning. Further integration of imaging and anatomical research will optimize clinical outcomes in procedures involving the face.
